# The dual effect of acetate on microglial TNF-α production

**DOI:** 10.1016/j.clinsp.2022.100062

**Published:** 2022-06-29

**Authors:** Matheus Garcia Fragas, Daniel May de Oliveira, Meire Ioshie Hiyane, Tarcio Teodoro Braga, Niels Olsen Saraiva Camara

**Affiliations:** a Department of Immunology, Instituto de Ciências Biomédicas (ICB IV), Universidade de São Paulo, São Paulo, SP, Brazil; b Department of Basic Pathology, Universidade Federal do Paraná, Curitiba, PR, Brazil; c Biosciences and Biotechnology Graduation Program, Instituto Carlos Chagas (ICC), Fiocruz, Curitiba, PR, Brazil; d Nephrology Division, Universidade Federal de São Paulo, São Paulo, SP, Brazil

**Keywords:** Acetate, Microglia, Inflammation, Alzheimer Disease, Encephalomyelitis, TNFa, Neuropharmacology

## Abstract

**Introduction:**

Short-Chain Fatty Acids (SCFA) are products of intestinal microbial metabolism that can reach the brain and alter microglia in health and disease contexts. However, data are conflicting on the effect of acetate, the most abundant SCFA in the blood, in these cells.

**Objective:**

The authors aimed to investigate acetate as a modulator of the inflammatory response in microglia stimulated with LPS.

**Method:**

The authors used an immortalized cell line, C8-B4, and primary cells for *in vitro* treatments with acetate and LPS. Cell viability was analyzed by MTT, cytokine by RT-PCR, ELISA, and flow cytometry. The authors also performed *in vivo* and *in silico* analyses to study the role of acetate and the TNF-α contribution to the development of Experimental Autoimmune Encephalomyelitis (EAE).

**Results:**

Acetate co-administered with LPS was able to exacerbate the production of pro-inflammatory cytokines at gene and protein levels in cell lines and primary culture of microglia. However, the same effects were not observed when acetate was administered alone or as pretreatment, prior to the LPS stimulus. Additionally, pharmacological inhibition of histone deacetylase concomitantly with acetate and LPS led to decreased TNF-α production. *In silico* analysis showed a crucial role of the TNF-α pathway in EAE development. Moreover, acetate administration *in vivo* during the initial phase of EAE led to a better disease outcome and reduced TNF-α production.

**Conclusion:**

Treatment with acetate was able to promote the production of TNF-α in a concomitant LPS stimulus of microglia. However, the immune modulation of microglia by acetate pretreatment may be a component in the generation of future therapies for neurodegenerative diseases.

## Highlights

• Acetate was able to exacerbate the production of TNF-α in microglia.
• Acetate administered as pre-treatment to LPS acts as an anti-inflammatory.
• Histone deacetylase decreased TNF-α production in Acetate- and LPS-treated cells.
• Depending on the time of administration, Acetate modulates microglia's activation.
• Acetate may threaten neurodegenerative and neuropsychiatric diseases.

## Introduction

Microglia are resident macrophages of the CNS, originating from primitive macrophage progenitors from the yolk sac, which migrate and then reside in the CNS. They spread rapidly through the brain and the spinal cord, where they form a self-renewable population without the need for myeloid recruitment during homeostasis.^1^, ^2^, ^3^ They are important in scavenging the brain parenchyma for pathogens and damage-related molecules through different receptors and are important in the synaptic remodeling and phagocytosis of cellular debris. Microglia can also initiate a neuroinflammatory response and recruit additional microglia as well as other immune cells.^2^^,^^4^, ^5^, ^6^, ^7^, ^8^

In many neurodegenerative diseases, microglial activation represents a key step to disease development such as Multiple Sclerosis, Alzheimer's disease, Parkinson's disease, and amyotrophic lateral sclerosis, and microglia modulation seems to have an impact on the treatment in disease models.^9^, ^10^, ^11^ Several studies have shown that different products of intestinal microbial metabolism can alter the profile of microglial activation in health and disease.^12^, ^13^, ^14^ Accordingly, Short-Chain Fatty Acids (SCFA) are products derived from the fermentation of indigestible dietary fibers carried out by the intestinal microbiota and can interact with immune cells, altering their function.^15^ Among them, acetate is the one that stands out with higher serum concentration and it functions as the substrate for acetyl coenzyme A synthesis and the regulation of gene expression via acetylation of histone.^16^^,^^17^ Additionally, SCFA has immunomodulatory, anti-proliferative and pro-apoptotic effects via activation of G-protein coupled receptors.^17^, ^18^, ^19^, ^20^ Alternatively, SCFAs can inhibit the activity of Histones Deacetylases (HDACs) via epigenetic control.^17^ Acetate also accentuates the DNA acetylation in the CNS through different mechanisms that vary in a dose and time-dependent way.^21^ Specifically, in microglia, the administration of acetate (*in vitro* and *in vivo*) prior to an inflammatory stimulus was able to reduce the release of IL-1b, IL-6, and TNF-α and increased histone acetylation.^22^, ^23^, ^24^

The result of this increased acetylation manifests itself through the attenuation of inflammatory parameters as the expression of proinflammatory cytokines of innate immunity.^22^ The final effect of this chain of events *in viv*o may be the preservation of neurons and a better clinical evolution in situations of neuroinflammation, as previously observed.^22^ Additionally, the authors investigated the SCFA, acetate, as a modulator of TNF-α and IL-6 in both LPS-activated primary and C8-B4 microglial cells at different administration schemes. The cell viability and the mechanism responsible for acetate modulation-either epigenetic or GRP receptor-activation were also explored.

## Results

### Effect of acetate on cytokine production

**Fig. 1:**
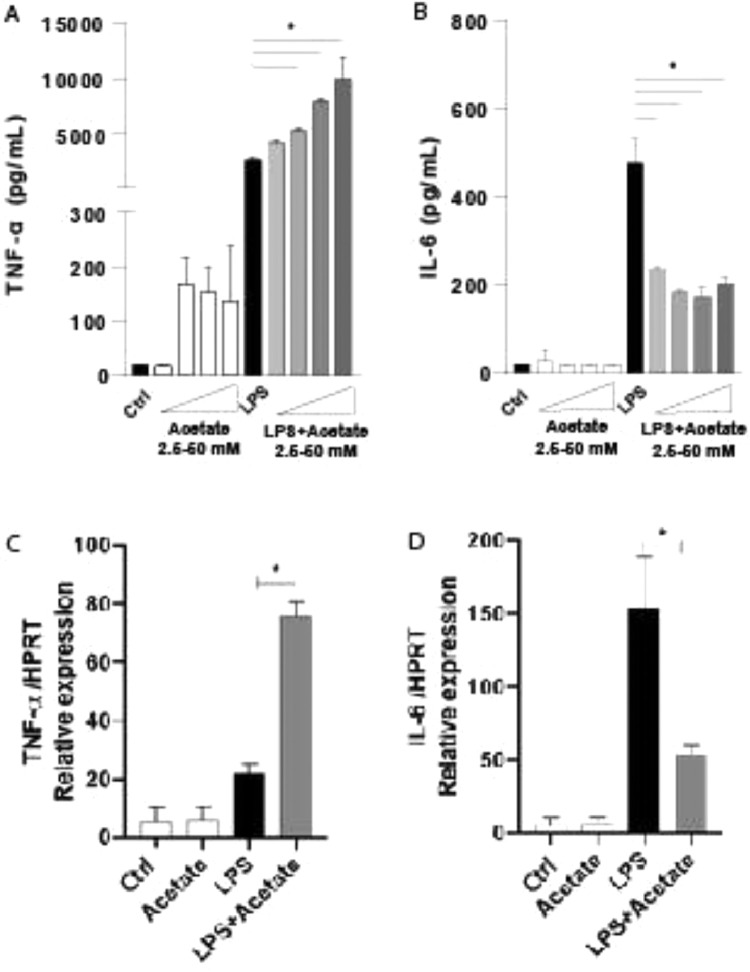
Effect of acetate on cytokine production. (A and B) Microglial C8-B4 cells were treated for 48 hours with acetate in different concentrations (2.5; 12.5; 25 and 50 mM), with LPS (0.25 µg/mL) or in combination. The supernatant was collected and concentrations of (A) TNF-α and (B) IL-6 were quantified via ELISA. (C and D) Microglial C8-B4 cells were treated for 6 h with acetate (25 mM), with LPS (0.25 µg/mL), or in combination, and the gene expression of (C) TNF-α and (D) IL-6 were analyzed by RT-PCR. Experiments were performed five times in triplicates. Data are presented as mean + SEM, *p < 0.05.

To understand the role of the acetate in the microglia modulation, the authors stimulated the C8-B4 microglial cells with acetate in the presence or absence of LPS at 0.25 µg/mL and the authors measured the TNF-α and IL-6 production within 48 hours (Fig. 1A and 1B). The TNF-α production increases in a dose-dependent response, being the higher dose of acetate, the higher TNF-α levels, but only in the presence of LPS (Fig. 1A). On the other hand, the production of IL-6, a pro-inflammatory-related cytokine,^25^ was significantly reduced no matter the dose of acetate, when compared to LPS alone (Fig. 1B). In an attempt to understand the cytokines kinetics production, the authors evaluated the gene expression in C8-B4 cells treated with acetate in the presence or absence of LPS for 6 hours. The authors observed that the inflammatory pattern followed by acetate stimulus remains in accordance with the protein levels of TNF-α and IL-6 (Fig. 1C and 1D), being the gene expression levels of TNF-α higher in the acetate treatment when compared to LPS alone. IL-6 acts in an opposite direction, with a reduction of its gene levels after treatment with acetate (Fig. 1D).

**Fig. 2:**
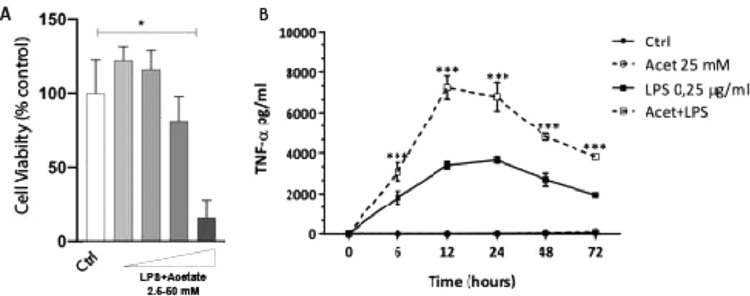
Effect of acetate on microglial cell viability and on TNF-α time course production. (A) Microglial C8-B4 cells were treated with acetate in different concentrations (2.5; 12.5; 25 and 50 mM) for 48 hours, and cell viability was analyzed by MTT assay. (B) Microglial C8-B4, untreated and treated with 25 mM acetate, 0.25 µg/mL LPS, or both, were maintained for 72 hours and TNF-α concentrations were analyzed at several points. Experiments were performed five times in triplicates. Data are presented as mean + SEM, *p < 0.05.

The authors next investigated whether the observed production of cytokines could be a response to cell death at different concentrations of acetate. Cells were treated with growing concentrations of acetate for 48 hours and cell viability was measured by the MTT (Fig. 2A). It is observed that acetate, at 50 mM reduced cell viability/proliferation. Moreover, concentrations of 12.5 and 25 mM showed no effects on cell viability/proliferation. Based on these results, the authors decided to use the 25 mM dose, the most effective in increasing the production of TNF-α with no effect on cell viability/proliferation. Additionally, a time-course production of TNF-α reveals a peak at 12 hours post-administration of acetate in the presence of LPS (Fig. 2B). Also, this production decreases over time, but it is still significantly higher at 72 hours post-administration when compared to the non-stimulated controls (Fig. 2B). It is noteworthy to say that acetate, which ate 25 mM and control did not increase TNF-α release (Fig. 2B).

**Fig. 3:**
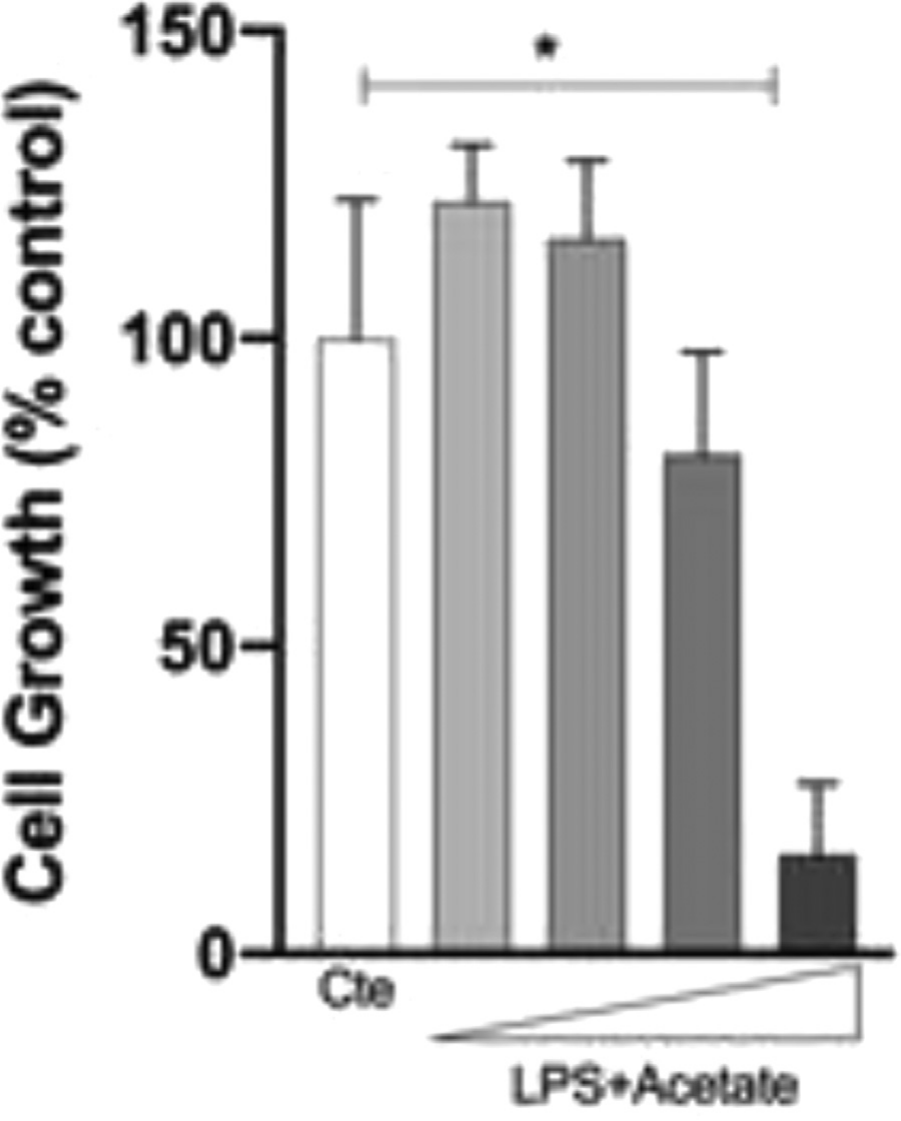
Production of TNF-α in primary microglia. Primary microglial cells were treated with acetate (25 mM), with LPS (0.25 µg/mL) alone or in combination of both for 24 hours and the percentage of cells expressing TNF-α were analyzed. In A, representative dot plots. In B, the quantification. Experiments were performed five times in triplicates. Data are presented as mean + SEM, *p < 0.05.

Despite C8-B4 cells representing a good *in vitro* modeling strategy for studying microglia biology,^26^ the authors argued whether they could indeed mimic primary microglia isolated from mice. For that, the authors isolated microglia from newborn mice's brains and treated them with growing concentrations of acetate in the presence of LPS for 24 h. Through flow cytometry, the authors observed that acetate was able to increase the amount of TNF-α in LPS-treated cells (Fig. 3), corroborating the previous results with C8-B4 cells and, therefore, confirming that the used cell lineage responds closely to primary microglia.

### Different time-related protocol of acetate stimulus leads to the different cytokines production profile

**Fig. 4:**
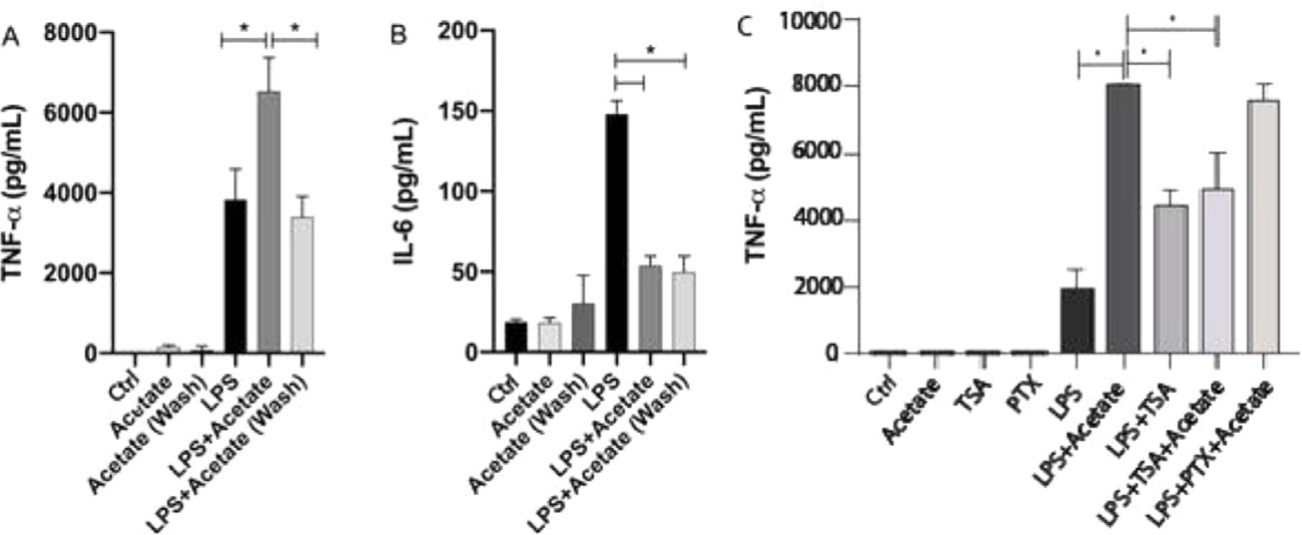
Acetate pre-treatment leads to reduced TNF-α production. (A and B) Microglial C8-B4 cells, untreated, treated with acetate (25 mM), with LPS (0.25 µg/mL), in combination, or acetate administered as a pre-treatment 24 h before LPS and washed (wash), were maintained for 48 h and (A) TNF-α and (B) IL-6 concentrations were analyzed in the supernatant. In C, C8-B4 cells were treated with acetate (25 mM), Trichostatin (TSA – 1 ng/mL), Pertussis toxin (PTX ‒ 100 ng/mL), LPS (0.25 µg/mL), or in combination with LPS and acetate. Experiments were performed five times in triplicates. Data are presented as mean + SEM, *p < 0.05.

The authors have additionally tested the effect of acetate in a “pre-treatment manner” in microglial cells. After 24 hours of pre-treatment, cells were washed and submitted to LPS stimulation for additional 48 hours. Surprisingly, this stimulation scheme was able to induce a significant reduction of TNF-α levels in relation to cells stimulated simultaneously with LPS and acetate (Fig. 4A). Although small, the pre-treatment of acetate was able to induce a significant reduction in the production of TNF-α in the group treated with LPS in comparison with just the LPS treatment (Fig. 4A). This data demonstrates the role of acetate as an anti-inflammatory modulator when administered previously to the LPS stimulus (Fig. 4A). Acetate pre-treatment, in turn, also led to reduced IL-6 production, consistent with the reduction observed in the concomitant LPS and acetate stimulation (Fig. 4B).

### Mechanism of acetate immunomodulation

The effect of acetate in microglia immunomodulation can be mediated via either the epigenetic control, Acting on Histone Acetyl Transferase (HAT) and Histone Deacetylase (HDAC),^15^ or via the activation of G protein-coupled receptors (GPR41 and GPR43). In order to evaluate the participation of these two mechanisms in the TNF-α, the production the authors used Trichostatin A (TSA), an inhibitor of HDAC,^27^ and Pertussis Toxin (PTX), an SCFA receptors’ (GPR41 and GPR43) blockers.^28^ The authors observed that treatment with 1 ng/mL TSA was able to increase the LPS-induced TNF-α secretion (Fig. 4C). Moreover, the TSA treatment led to a decrease in the TNFa production in comparison with the LPS+Acetate treated group indicating that both acetate and TSA may compete for histone acetylation. On the other hand, PTX presented no significant effect on TNF-α production in acetate- and LPS-stimulated cells (Fig. 4C). Cell behavior followed by TSA or PTX stimuli onto TNFα secretion, suggests that acetate can be epigenetically mediated, *i.e.*, by inhibition of HDACs.

### Effect of acetate the development of EAE

**Fig. 5:**
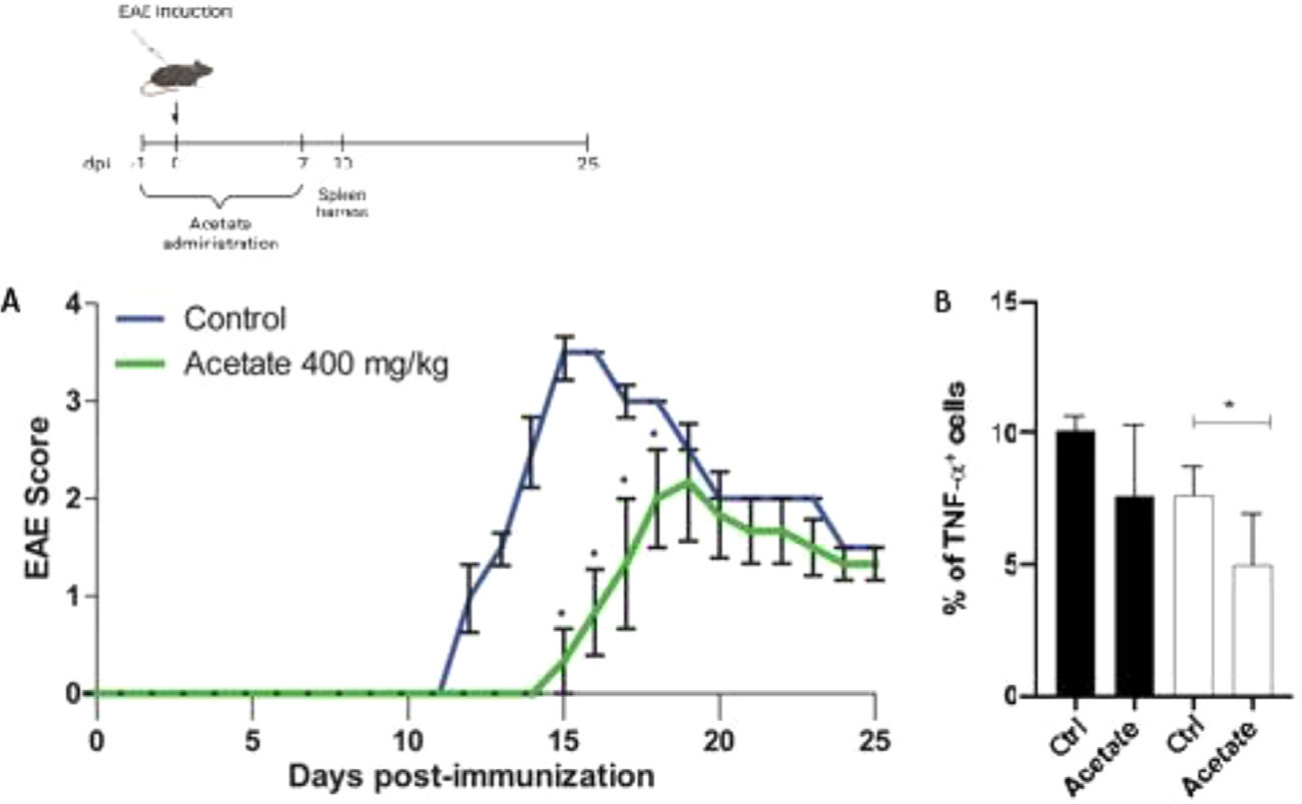
Effect of Acetate in the development of EAE. (Upper part) Schematic representation of the EAE experimental design. (A) C57Bl6 control and acetate treated (40 mg/kg – from day −1 to day 8) animals were immunized with MOG_35–55_ and monitored daily for 25 days to evaluate the clinical evolution of EAE. In B, animals were euthanized at day 10 post immunization and the percentage of TNF-α^+^ cells were evaluated in splenocytes. Cells were treated (black bars) or not (white bars) with PMA (Phorbol myristate acetate) and ionomycin for 24 hours. Experiments were performed twice. *N* = 5 per experiment. Data are presented as mean + SEM, *p < 0.05.

In order to investigate the anti-inflammatory effects of acetate in a pre-treatment manner *in vivo*, the authors proceeded to the Experimental Autoimmune Encephalomyelitis (EAE), mice were treated with vehicle or acetate (400 mg/kg) for 8 days (−1 to day 7). The treatment with acetate or vehicle was finished before the appearance of the first's symptoms, commonly present after the 7 to 10th-day post-immunization, characteristic of the induction phase of the disease, thus being a result comparable to the pre-treatment *in vitro*. The development of the disease was delayed in the group treated with acetate compared to the vehicle group (control) as shown in Fig. 5A. The authors observed the same profile of the *in vitro* findings, where the acetate acts in an anti-inflammatory way when administered before the inflammatory stimulus. Additionally, acetate-treated mice had milder clinical EAE scores in the comparison with control mice (Fig. 5A). The authors further evaluated the TNF-α production from both animals. Similarly, as seen in the *in vitro* data, cells isolated from acetate-treated animals subjected to EAE show less expression of TNF-α, demonstrating that the treatment is capable of inducing long-term anti-inflammatory effects, even after the cells were removed from the organisms (Fig. 5B).

### In silico analysis of spinal cords of EAE mice

**Fig. 6:**
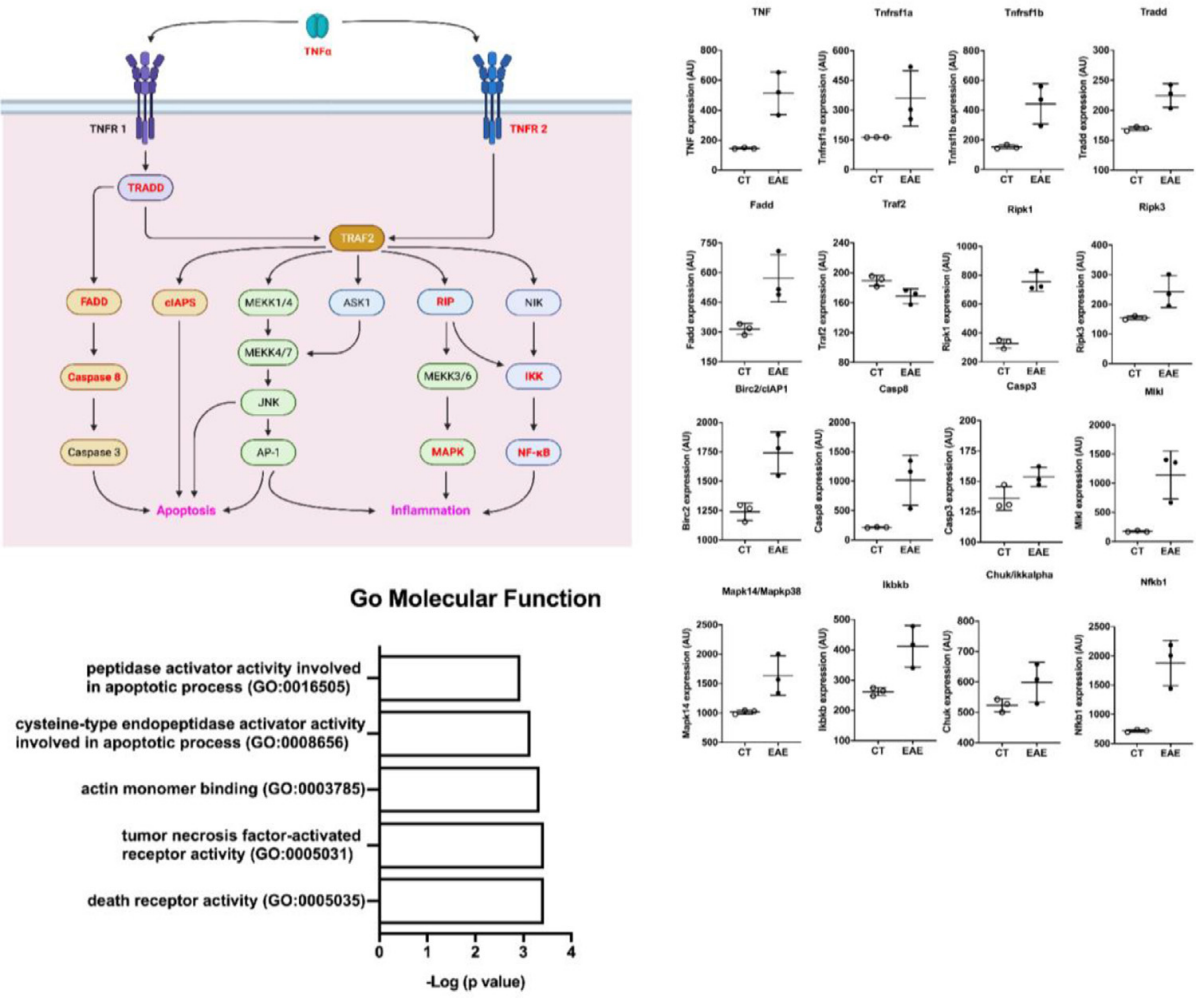
In silico analysis of spinal cords of EAE mice. (A) Scheme of TNF-α pathway activation. Genes upregulated in the dataset are shown in red. (B) The differential expression of genes related to the scheme seen in A. (C) Pathway analysis of the dataset where the Gene Ontology Molecular Function is shown.

Trying to understand the relevance of TNF-α and its activation pathway, the authors performed *in silico* analysis from public microarray data of the spinal cord of animals submitted to EAE and their healthy controls (Fig. 6) (GSE60847). A schematic view of the TNF-α contribution to the EAE development was generated (Fig. 6a). The differential expression analysis showed an increase in the expression of genes related to the TNF-α pathway in EAE animals, including TNF, TNF receptors, adaptor proteins, caspases, MAPK, and NF-kb (Fig. 6b). This shows the important role of TNF-α in the pathology of EAE and inflammation. In addition, pathway enrichment analysis based on the 500 up-regulated genes in the Gene Ontology (molecular function) shows that activity related to TNF-α and its receptors was important in the spinal cord of mice with EAE (Fig. 6c).

Altogether, TNF-α presents an essential role in the generation of neuroinflammation and the immune modulation of microglia by acetate may be a component in the generation of future therapies for neurodegenerative diseases, as long as the time for starting the treatment is taken into account.

## Discussion

Microglia continually monitor the tissue parenchyma for pathological alterations, under steady-state, and play important roles in the regulation of CNS synaptic pruning. In the occurrence of an inflammatory insult, microglia, and CNS-infiltrating macrophages not only constitute the first line of defense against pathogens by regulating components of innate immunity, but they also regulate the adaptive immune responses. Dysregulation of the microglial development and function impacts both health and disease status.^2^^,^^7^^,^^29^^,^^30^

It is known that peripheral and systemic changes are capable of altering the pathophysiology of the CNS, including products derived from the gut microbiota.^31^ Specifically, microglia seem to need signals derived from the microbiota for maturation and adequate function.^32^ SCFAs mediate the function of immune cells, both intestinal and systemic, and even in brain cells since they can cross the blood-brain barrier, and changes in SCFA concentrations appear to be related to CNS pathologies.^31^ In the present study, the authors show that acetate affects the release of TNF-α in a dose and time-dependent way. However, acetate alone was not able to induce this proinflammatory cytokine, which suggests the participation of acetate in exacerbating the response against LPS. It is noteworthy to say that the C8-B4 cell line, analyzed in the present study, has been obtained from a newborn's brain, and it is already known that microglial heterogeneity changes throughout the lifespan and brain area.^32^

Additionally, it is still unclear how SCFAs participate in the immune modulation since conflicting results have been recorded in the literature. Butyrate, for example, one of the most studied SCFAs, has shown a pro-inflammatory and anti-inflammatory capacity in different diseases of the SNC.^31^^,^^33^^,^^34^ The same has been observed for other SCFA, as results show their role in the development of neurodegenerative diseases,^31^^,^^34^^,^^35^ while other studies demonstrate their participation in the worsening of the neuroinflammatory condition.^33^^,^^36^^,^^37^ Some studies have shown the ability of acetate to activate immune cells.^38^, ^39^, ^40^, ^41^ Despite some reports pointing to SCFAs as exerting suppressive effects on the activation of immune cells,^15^^,^^18^^,^^20^^,^^42^ the present data demonstrate increased TNF-α production, at the protein and gene transcription levels, upon acetate stimulation on LPS-treated cells. Such discrepancy could be attributed to different cell lines used in the different studies. Moreover, it is also demonstrated in the literature that acetate leads to the production of Reactive Oxygen Species (ROS) in neutrophils^38^ and macrophages,^39^ which ultimately leads to the production of TNF-α, perpetuating the inflammatory state.^43^

Bearing in mind that microglia activation is related to several neuropathologies, including Alzheimer's Disease and Multiple Sclerosis (MS), and that TNF-α production is present as an activation trigger, the authors decided to investigate whether acetate is capable of inducing long-lasting effects, even before an inflammatory insult. The levels of TNF-α have been reported to be severely high in the gray matter of EAE and MS brains, pointing to TNF-α as a primary neurotoxic molecule in EAE, as confirmed by the present *in-silico* analysis. It has been shown stronger pro-inflammatory responses of immune cells and their associated cytokines, chemokines, and receptors in males *vs*. female,^44^ which can represent a caveat in the present study. Additionally, up-regulation of TNF-α is followed by increased Th17 immune response and an exacerbated EAE-related disease. Surprisingly, when acetate was administered before LPS, instead of concurrently, the inflammatory effect of acetate was not observed, reflected by diminished levels of TNF-α. This data indicates that acetate affects microglia immediately, suggesting a rapid interaction with receptors or even a mediation through epigenetic modification. The data on GPR blockade and HDAC inhibition indicate that TNF-α production by microglia is due to histone acetylation, ultimately supporting gene transcription thorough upon cellular activation with LPS. Interestingly, the authors observed that acetate acts in antagonistic ways depending on the time of administration. In concomitant treatment with LPS, the authors observed an increase in TNF-α, however, both *in vitro* and *in vivo* data demonstrate that acetate acts in an anti-inflammatory way as pre-treatment. Here the authors clarify the inconsistencies in the literature and prove that acetate, depending on the time of administration, can act in antagonistic ways in the cellular inflammatory profile.

Altogether, the present study's data demonstrate that acetate can promote chromatin accessibility, histone acetylation, and TNF-α production in LPS-stimulated microglia. The use of acetate was explored in the context of tumors, in which it was demonstrated that acetate enhances IFN-g gene transcription^43^ in addition to presenting effects on T-cell effector function after prolonged glucose restriction.^45^ The authors believe that a better understanding of the role of SCFA and its immune modulation may be an important component in the generation of future therapies for neurodegenerative diseases. Depending on the time of administration, acetate can modulate the activation of microglia.

## Material and methods

### Cell culture and treatment

The murine microglial cell line C8-B4 (ATTC) was cultured in Dulbecco's Modified Eagle Medium: Nutrient Mixture F-12 (Gibco™) supplemented with 10% fetal bovine serum (Sigma-Aldrich), 100 U/mL penicillin and 100 μg/mL streptomycin (Life Technologies), at 37 °C in 5% CO_2_. Acetate solution was prepared using acetic acid glacial (Labsynth™). For use, it was diluted to 1 Molar stock solution, pH = 7.4. Used concentrations were 2.5; 12.5; 25; and 50 mM, according to the figure. Primary mixed glial cells were cultured as described previously.^46^, ^47^, ^48^ Briefly, they were prepared from the brains of 0–2-day-old mice, C57Bl/6 J. Whole brains were dissected into the complete medium. Meninges were removed and cells dissociated by trituration prior to seeding at 5 × 10^5 cells/mL onto 175-cm² tissue culture flasks. The culture medium was changed weekly until the culture reached confluency (14–20 days).

Microglial cells were harvested from 175 cm^2^ flasks of mixed glial cultures by shaking at 245 r.p.m. for 2 h. Cells were collected by centrifugation and seeded at 5 × 10^5 cells/mL. After 1-hour of incubation at 37 °C, non-adherent or weakly adherent cells were removed by gentle shaking and washed out. Cells were further cultured in DMEM supplemented with 10% FBS for 1 day.

The reagents, LPS from Salmonella enterica (L5886) are used at a concentration of 0.25 µg/mL, Trichostatin A (T8552) at 1 ng/mL, and Pertussis toxin (P7208) at 100 ng/mL, all were purchased from Sigma-Aldrich.

### EAE model

Experimental protocols were reviewed and approved by the Institutional Animal Care and Use Committee (CEUA number 34, 100/12) in compliance with the Ethical Principles in Animal Research of the Brazilian College of Animal Experimentation. Mice were housed on a 12/12 h light/dark cycle with free access to chow and water in the Animal Facilities of the Department of Immunology, ICB, University of Sao Paulo. Within 2 days of life, the brain was extracted from animals of both sexes for the isolation and mixed culture of glial cells.

Female C57BL/6 (8‒12 weeks old) were immunized subcutaneously with MOG_35–55_ (150 μg) emulsified in CFA, with 500 μg of *M. tuberculosis* Des, H37Ra (Becton & Dickinson ‒ BD). They also received 2 doses of *Bordetella pertussis* toxin (200 ng) intraperitoneally, at 0 and 48 h after immunization. The animals were observed daily, and the scores were given as stated: 0-No disease, 1-Limp tail, 2-Weak/partially paralyzed hind legs, 3-Completely paralyzed hind legs, 4-Complete hind, and partial front leg paralysis, 5-Complete paralysis/death. The animals were followed for 25 days post-immunization for the disease progression, for the cytokine production, splenocytes, and the whole brain were isolated on the 10th-day post-immunization, and the intracellular staining was performed.

### Cells viability by mtt assay

The MTT (Thiazolyl Blue Tetrazolium Bromide) assay was carried out to determine the optimum concentration of acetate. Cells were seeded at 1.5 × 10^4 per well in a 96 well plate and, after 24 hours, the complete medium was replaced with a medium without FBS. 5 mg/mL of MTT was added to each well and incubated for 2 hours at 37 °C in 5% CO_2_, protected from light. The MTT reagent was removed and 100 μL of DMSO was added. The optical density was read at 570 nm with the Versamax Tunable Microplate Reader, Molecular Devices (Sunnyvale, California).

### Quantitative PCR

Total RNA from cells was extracted with Trizol® (Invitrogen, Carlsbad, CA). cDNA (2 µg of total RNA) was synthesized with Moloney Murine Leukemia Virus Reverse Transcriptase (Promega, Madison, WI, USA). The real-time PCR assay was carried out using 3 μL of cDNA as the template and 5 μL of Power SYBR Green PCR Master Mix (Thermo Fisher Scientific, Waltham, MA, USA). Primers used in this study are as followed, HPRT: 5′-CTCATGGACTGATTATGGACAGGA-3′ (F), 5′GCAGGTCAGCAAAGAACTTATAGCC-3′ (R); TNF-α: 5′-CATCTTCTCAAAATTCGAGTGACAA-3′ (F) 5′-TGGGAGTAGACAAGGTACAACCC-3′(R); IL6: 5′-CCGGAGAGGAGACTTCACAG-3′ (F) 5′-ACAGTGCATCATCGCTGTTC-3′ (R).

### Determination of cytokines by ELISA

TNF-α and IL-6 levels in the culture supernatants were measured using commercial sandwich ELISA kits (R&D Systems, Minneapolis, MN, USA) according to manufacturers’ instructions. Absorbance was measured at 450 nm, and the results are presented in ρg/mL.

### Flow cytometry

After the stimulus with LPS and acetate for 24 h, the microglial cells proceeded to flow cytometry. Cells were incubated for CD11b (pacific blue, BioLegend-San Diego, Ca, USA. cat: 101,224) with a concentration of 1: 100 for 30 minutes. For the analysis of the intracellular TNF-α production, the monoclonal anti-TNF-α antibody was used (BD Diagnostics®, Franklin Lakes, USA ‒ cat: 554,420). The acquisition and analysis of the samples were performed in a FACS-calibur flow cytometer (Becton & Dickinson, Mountain View, CA), using the CellQuest software (Apple).

### In silico analysis

The authors analyzed the expression of genes related to the TNF-α pathway through GEO2R (https://www.ncbi.nlm.nih.gov/geo/geo2r/) in a public dataset (GSE60847) and compared the differential expression of spinal cord genes between EAE and sham animals, the authors use only differentially expressed genes with adjusted *p* < 0.05. The 500 up-regulated genes were submitted to pathway enrichment using Enrichr (https://maayanlab.cloud/Enrichr/) and GO (https://geneontology.org).

### Statistics

Experiments were performed in triplicate and three independent tests were performed for each assay. The data were described in terms of the mean and S.E.M. unless specified in the figure legend. Differences between groups were compared using ANOVA (with Tukey's post-test) and Student's *t*-test. A 95% significance level was used, and differences were regarded as *p* < 0.05. Statistical analyses were performed using GraphPad PRISM 6.01 (La Jolla, CA, USA). No masking and no blinding were used during group allocation.

## Authors' contributions

M.G.F. and D.M.O performed all the experiments analysis; M.I.H. gave technical support; T.T.B. contributed to the data analysis and wrote the manuscript; N.O.S.C. provided the financial support and supervised the students.

## Funding

This study was supported by the São Paulo State Funding Agency (FAPESP) (grants numbers: 17/05,264–7), Conselho Nacional de Desenvolvimento Científico e Tecnológico (CNPq) and in part by the Coordenação de Aperfeiçoamento de Pessoal de Nível Superior ‒Brasil (CAPES) Financial Code 001.

## Conflicts of interest

The authors declare no conflicts of interest.
